# Improvements in six aspects of quality of care of incident hemodialysis patients – a real-world experience

**DOI:** 10.1186/s12882-021-02529-1

**Published:** 2021-10-07

**Authors:** Maciej Drozdz, João Frazão, Fatima Silva, Partha Das, Werner Kleophas, Wisam Al Badr, Szymon Brzosko, Stefan H. Jacobson

**Affiliations:** 1 DaVita Poland, Kraków, Poland; 2 DaVita Portugal, Lisbon, Portugal; 3 Department of Nephrology, São João Hospital Center, Porto, Portugal; 4 School of Medicine, University of Porto, Porto, Portugal; 5 DaVita International, London, UK; 6 King’s College Hospital NHS Foundation Trust, London, UK; 7 DaVita Germany, Düsseldorf, Germany; 8 Clinic for Nephrology, Heinrich-Heine University, Düsseldorf, Germany; 9 DaVita Kingdom of Saudi Arabia, Riyadh, Saudi Arabia; 10 1st Department of Nephrology and Transplantation, Medical University of Bialystok, Białystok, Poland; 11 DaVita Poland, Wroclaw, Poland; 12 Department of Nephrology, Department of Clinical Sciences, Karolinska Institutet, Danderyd University Hospital, Stockholm, Sweden

## Abstract

**Background:**

The transition from chronic kidney disease stage 5 to initiation of hemodialysis has gained increased attention in recent years as this period is one of high risk for patients with an annual mortality rate exceeding 20%. Morbidity and mortality in incident hemodialysis patients are partially attributed to failure to attain guideline-based targets. This study focuses on improvements in six aspects of quality of dialysis care (adequacy, anemia, nutrition, chronic kidney disease-mineral bone disorder (CKD-MBD), blood pressure and vascular access) aligning with KDIGO guidelines, during the first 6 months of hemodialysis.

**Methods:**

We analyzed patient demographics, practice patterns and laboratory data in all 3 462 patients (mean age 65.9 years, 41% females) on hemodialysis (incident <90 days on hemodialysis, *n*=603, prevalent ≥90 days on hemodialysis, mean 55 months, *n*=2 859) from all 56 DaVita centers in Poland (51 centers) and Portugal (5 centers). 80% of patients had hemodialysis and 20% hemodiafiltration. Statistical analyses included unpaired and paired Students t-test, Chi-2 analyses, McNemar test and logistic regression analysis.

**Results:**

Incident patients had lower Kt/V (1.4 vs 1.7, *p*<0.001), lower serum albumin (37 vs 40 g/l, *p*=0.001), lower Hb (9.9 vs 11.0 g/dl, *p*<0.001), lower TSAT (26 vs 31%, *p*<0.001), lower iPTH (372 vs 496 pg/ml, *p*<0.001), more often a central venous catheter (68 vs 26%, *p*<0.001), less often an AV fistula (34 vs 70 %, *p*<0.001) compared with all prevalent patients. Significantly more prevalent patients achieved international treatment targets.

Improvements in quality of care was also analyzed in a subgroup of 258 incident patients who were followed prospectively for 6 months. We observed significant improvements in Kt/V (*p*<0.001), albumin (*p*<0.001), Hb (*p*<0.001) transferrin saturation (TSAT, *p*<0.001), iPTH (*p*=0.005) and an increased use of AV fistula (*p*<0.001). Furthermore, logistic regression analyses identified treatment time and TSAT as major factors influencing the attainment of adequacy and anemia treatment targets.

**Conclusion:**

This large real-world European multicenter analysis of representative incident hemodialysis patients indicates that the use of medical protocols and medical targets assures significant improvements in quality of care, which may correspond to better outcomes. A selection bias of survivors with less comorbidities in prevalent patients may have influenced the results.

## Background

The transition from chronic kidney disease stage 5 to initiation of hemodialysis has received increased attention in recent years, as this period is one of high vulnerability for patients, with an annual mortality rate exceeding 20% [1–3]. The increased risk of mortality in incident patients is associated with the presence of risk factors such as the quality of pre-dialysis care, age, gender and concurrent comorbidities, for instance diabetes mellitus, cardiovascular and nutritional status [1–3].

Over the past decades, there has been a tendency towards commencement of hemodialysis at higher levels of residual kidney function [4–7]. Definitive clinical trials of early versus later timed hemodialysis initiation have been difficult to conduct, partly due to the unpredictable clinical course that often accompanies deterioration in renal function. Still after the Initiating Dialysis Early and Late (IDEAL) trial [8], substantial debate continues regarding the impact on mortality and outcomes of initiating dialysis early versus late [4–7].

Previous studies have shown that the risk of early mortality after starting hemodialysis is independently associated with failure to attain guideline-based international treatment targets for dialysis adequacy (dose of hemodialysis, Kt/V and urea reduction ratio, URR), nutrition (serum albumin), renal anemia (hemoglobin and iron levels), CKD-MBD (serum phosphorus, calcium, PTH) and blood pressure [9–12]. Early mortality risk is also related to features of predialysis care which includes delayed referral to nephrology services and having an arteriovenous fistula or a central venous catheter as primary vascular access when commencing hemodialysis [13–15].

The importance of providing individual patients adequate quality of care before and following start of hemodialysis is well recognized. Identifying modifiable treatment factors that are associated with the heightened risk of morbidity and mortality during this early dialysis period is thus essential.

This large European multicenter study included all patients treated at all DaVita dialysis facilities in Poland and Portugal and focuses on alignment to international guidelines and improvements in six aspects of quality of hemodialysis care: dialysis adequacy, renal anemia, nutrition, CKD-MBD, blood pressure control and use of vascular access in patients on maintenance hemodialysis. We measured alignment with treatment targets monthly and used medical protocols and systematic follow up of the accomplishment of targets in all incident patients during the first six months of hemodialysis.

## Methods

### Patients and data collection

We analyzed patient demographics, hemodialysis practice patterns and laboratory data from all 3 462 patients (mean age 65.9 years, 41% females) on hemodialysis (incident <90 days on hemodialysis, *n*=603, prevalent ≥90 days on hemodialysis, mean hemodialysis vintage 55 months, *n*=2 859 patients) from all 56 DaVita hemodialysis facilities in Poland (51 centers) and Portugal (5 centers). Eighty percent of patients had been treated by hemodialysis and 20% hemodiafiltration. Since *all* patients treated at *all* dialysis facilities were included in the analysis, this cohort represented a “real-world” clinical experience.

In all facilities, blood samples were collected monthly or quarterly in accordance with international dialysis guidelines (European Renal Best Practice (ERBG) guidelines and KDIGO (Kidney Disease Outcomes Quality Initiative) guidelines. All clinics followed medical protocols and aimed the same medical targets being representative for abovementioned guidelines. Patient and treatment characteristics and biochemical data were collected during routine hemodialysis practice. Demographic and laboratory data, as well as information on practices, were analyzed in all patients in the same month of 2019. Laboratory analyses were made at local laboratories with validated and recommended procedures. Kt/V was assessed as single pool Kt/V (spKt/V) and intact PTH (iPTH) was measured. All patients treated with erythropoiesis stimulating agents (ESA) had erythropoietin alpha or beta. The erythropoiesis-stimulating agent resistance index (ERI) was defined as the weight-adjusted weekly ESA dose divided by the hemoglobin value (IU/week/kg/Hb).

### Statistical analyses

Statistical analyses were performed using IBM SPSS Statistics version 25. All values are presented as mean or standard deviation (SD) or proportions and counts. We compared all incident to all prevalent patients (unpaired Students t-test and Chi-2 analyses) and in addition analyzed improvements in six aspects of quality of dialysis care in a subgroup of patients who were followed prospectively for 6 months (paired Students t-test and McNemar analyses). Logistic regression analysis was done to identify factors significantly influencing the achievement of treatment targets. A *p*-value <0.05 was considered statistically significant.

## Results

### Incident vs prevalent patients on hemodialysis

We analyzed six aspects of quality of hemodialysis care (adequacy, nutrition, renal anemia, CKD-MBD, blood pressure control and vascular access) in 603 patients on hemodialysis for less than 90 days (incident patients) and compared the results to 2 859 patients who had been on maintenance hemodialysis for more than 90 days (prevalent patients, Table 1). There were no significant differences in age, gender or Charlson comorbidity index between the groups (Table 1). Diabetes was more common in prevalent (27%) than in incident patients (23%, *p*<0.05). In addition, more prevalent patients had been treated by hemodiafiltration (22% vs 11%).

**Table 1:** Comparison of six aspects of quality of care in 603 incident patients (<90 days on hemodialysis) and 2 859 prevalent patients (>90 days on maintenance hemodialysis)

Aspects of quality of care	Incident patients *n*=603	Prevalent patients *n*=2859	p*
	Mean (SD)	Mean (SD)	t-test
Age (years)	66.3 (14.6)	67.1 (14.2)	NS
Charlson comorbidity index	6.9 (2.9)	6.9 (2.9)	NS
**Adequacy**			
Weekly treatment time (min)	677 (116)	721 (77)	<0.001
Blood flow rate (ml/min)	295 (65)	351 (77)	<0.001
Treated blood volume (l/kg)	1.0 (0.3)	1.2 (0.7)	<0.001
HD treatments/week	3.0 (0.4)	3.0 (0.3)	NS
Kt/V	1.4 (0.4)	1.7 (0.4)	<0.001
URR (%)	66 (16)	74 (10)	<0.001
Potassium (mmol/l)	4.8 (0.7)	5.2 (1.0)	<0.001
**Nutrition**			
Body weight	70.8 (20)	72.4 (17)	NS
BMI (kg/m^2^)	25.9 (0.9)	26.3 (6.5)	NS
Albumin (g/l)	37.4 (19.5)	40.7 (21.8)	0.001
**Anemia**			
Hb (g/dl) all pts	9.9 (1.6)	11.0 (1.3)	<0.001
Hb (g/dl) on ESA	9.8 (1.5)	10.8 (1.2)	<0.001
TSAT (%)	26 (12)	31 (14)	<0.001
Ferritin (μg/l)	305 (291)	540 (542)	<0.001
Weekly ESA dose (IU/week)	5470 (4440)	4982 (3640)	<0.05
ERI (ESA U/week/kg/Hb)	8.5 (8.4)	7.0 (6.3)	<0.001
**CKD MBD**			
Phosphorus (mg/dl)	4.8 (1.6)	5.0 (1.6)	NS
Calcium (mg/dl)	8.7 (2.2)	8.8 (1.6)	NS
iPTH (pg/ml)	372 (298)	496 (448)	<0.001
**Blood pressure**			
MAP (mm Hg)	95 (12)	94 (14)	NS
IDBWG (%)	1.6 (4.6)	2.6 (1.5)	<0.001
UF volume (ml/session)	1687 (963)	2259 (1008)	<0.001
**Vascular access**	Percent	Percent	Chi square
AV fistula (%)	32	70	<0.001
AV graft (%)	1	4	<0.001
CVC (%)	68	26	<0.001
Sex (% female)	42	41	NS
Age ≥70 years (%)	46	47	NS
Diabetes mellitus (%)	23	27	<0.05
Hemodiafiltration (%)	11	22	<0.001

*Abbreviations*: *HD* hemodialysis, *URR* urea reduction ratio, *BMI* body mass index, *ESA* erythropoiesis stimulating agent, *TSAT* transferrin saturation, *ERI* erythropoietin resistance index, *iPTH* intact parathyroid hormone, *MAP* mean arterial pressure, *IDBWG* interdialytic body weight gain, *AV* arteriovenous, *CVC* central vascular catheter

* Students t-test and Chi-square analysis for continuous numbers (mean and SD) and percentages respectively

Prevalent patients had higher Kt/V (1.7 vs 1. 4, (*p*<0.001), longer treatment time, higher blood flow rate and consequently a higher treated blood volume per session than incident patients (*p*<0.001 for all comparisons, Table 1). Furthermore, serum albumin and potassium were higher (*p*<0.001) in prevalent patients, maybe indicating a more favorable alimentary status. In terms of anemia control both all prevalent patients, and the subgroup of patients on ESA, had higher Hb, higher TSAT and ferritin than incident patients (*p*<0.001 for all comparisons, Table 1). Furthermore, the weekly ESA dose was lower and ERI lower in prevalent patients (*p*<0.05 and *p*<0.001 respectively). There were no significant differences in calcium and phosphorus concentrations between the groups, but iPTH was higher in prevalent compared to incident patients (*p*<0.001). Interdialytic body weight gain (IDWG) and ultrafiltration (UF) volume were both higher in prevalent patients (*p*<0.001) but MAP was similar in both groups. Prevalent patients more often had an AV fistula (70%) than incident patients (32%, *p*<0.001) and consequently the use of a central venous catheter was higher in incident (68%) compared to prevalent patients (26%, *p*<0.001, Table 1).

### Achievement of treatment targets

The attainment of treatment targets in international guidelines, in incident and prevalent patients is presented in Table 2. Significantly more prevalent hemodialysis patients had Kt/V ≥1.2, serum albumin ≥40 g/l, Hb 10-12 g/dl, TSAT≥20% and ferritin ≥200 μg/l (*p*<0.001 for all comparisons). We observed a small but significant difference in the control of iPTH between groups (Table 2).

**Table 2:** Achievement of international treatment targets in 603 incident patients (<90 days on hemodialysis) and 2859 prevalent patients (>90 days on maintenance hemodialysis)

Aspects of quality of care	Incident patients *n*=603	Prevalent patients *n*=2859	p*
	Percent	Percent	Chi square
**Adequacy**			
Weekly treatment time ≥720 min	68	87	<0.001
Blood flow rate ≥300 ml/min	67	96	<0.001
Kt/V ≥1.2	69	94	<0.001
Treated blood volume ≥ 1 l/kg	42	75	<0.001
**Nutrition**			
Albumin < 40 g/l	71	47	
≥40 g/l	29	53	<0.001
**Anemia**			
Hb <10 mg/dl	52	16	
Hb 10-12 mg/dl	41	67	<0.001
Hb >12 mg/dl	7	17	
TSAT ≥20%	66	82	<0.001
Ferritin ≥200 μg/l	55	81	<0.001
**CKD MBD**			
PTH <150 pg/ml	21	16	
PTH 150-600 pg/ml	62	57	<0.001
PTH >600 pg/ml	18	27	

* Chi-square analysis

### Improvements in quality of hemodialysis care in patients after 6 months of care

Improvements in six aspects of quality of hemodialysis care in 258 incident patients is presented in Table 3. We observed a significant improvement in Kt/V, increased treatment time, blood flow rate and treated blood volume over time (*p*<0.001 for all comparisons) in incident patients vs the same patients after 6 months of treatment at DaVita facilities. Hb increased with improved control of iron parameters (*p*<0.001) and a concomitant reduction of ESA dose (*p*=0.09) in combination with lower ERI (*p*=0.05). Small and statistically significant changes in serum calcium and iPTH were observed. Significantly more patients had an AV fistula after 6 months (55%, *p*<0.001) and fewer patients had a central venous catheter (42%, *p*<0.001) compared to incident patients.

**Table 3:** Improvement in six aspects of quality of hemodialysis care in incident patients (<90 days on hemodialysis) vs the same patients after 6 months of hemodialysis (*n*=258)

Aspects of quality of care	Incident patients (<90 days) *n*=258	Prevalent patients (>6 months) *n*=258	p*
Charlson comorbidity index	6.4 (2.7)	6.5 (2.6)	<0.01
**Adequacy**			Paired t-test
Weekly treatment time (min)	678 (122)	715 (87)	<0.001
Blood flow rate (ml/min)	294 (67)	329 (48)	<0.001
Treated blood volume (l/kg)	1.0 (0.3)	1.1 (0.3)	<0.001
HD treatments/week	3.0 (0.4)	3.0 (0.3)	NS
Kt/V	1.4 (0.5)	1.5 (0.4)	<0.001
URR (%)	66 (17)	72 (9)	<0.001
Potassium (mmol/l)	4.8 (0.7)	5.0 (0.7)	<0.01
**Nutrition**			
Body weight	70.8 (20)	71.6 (18.6)	NS
BMI (kg/m^2^)	25.9 (6.5)	25.9 (5.8)	NS
Albumin (g/l)	36.6 (4.6)	42 (3.4)	<0.01
**Anemia**			
Hb (g/dl) all pts	9.8 (1.5)	10.7 (1.4)	<0.001
Hb (g/dl) on ESA	9.8 (1.4)	10.6 (1.3)	<0.001
TSAT (%)	26 (12)	30 (13)	0.001
Ferritin (μg/l)	331 (330)	458 (507)	<0.001
Weekly ESA dose (IU/week)	5243 (4221)	4627 (4347)	0.092
ERI (EPO IU/week/kg/Hb)	8.2 (7.3)	5.5 (5.8)	0.05
**CKD MBD**			
Phosphorus (mg/dl)	4.8 (1.6)	5.0 (1.7)	0.084
Calcium (mg/dl)	8.5 (1.3)	8.6 (1.2)	<0.05
iPTH (pg/ml)	389 (316)	348 (305)	0.005
**Blood pressure**			
MAP (mm Hg)	95 (12)	95 (12)	NS
IDBWG (%)	1.8 (1.3)	1.9 (6.0)	NS
UF volume (ml/session)	1611 (934)	1964 (986)	<0.001
**Vascular access**	Percent	Percent	McNemars test
AV fistula (%)	35	55	<0.001
AV graft (%)	1	2	NS
CVC (%)	64	42	<0.001

*Abbreviations*: *HD* hemodialysis, *URR* urea reduction ratio, *BMI* body mass index, *ESA* erythropoiesis stimulating agent, *TSAT* transferrin saturation, *ERI* erythropoietin resistance index, *iPTH* intact parathyroid hormone, *MAP* mean arterial pressure, *IDBWG* interdialytic body weight gain, *AV* arteriovenous, *CVC* central vascular catheter

* Students t-test and McNemars test for continuous numbers and percentages respectively

### Improvements in the achievement of treatment targets over 6 months

We observed a significant improvement in the attainment of international treatment targets in terms of dialysis adequacy (*p*<0.001), nutrition (*p*<0.001) and control of renal anemia (*p*<0.001 both for all patients, and for patients on ESA) in patients treated for 6 months compared to the same patients during their first 90 days of hemodialysis care (Table 4).

**Table 4:** Improvement in the achievement of international treatment targets in 258 incident hemodialysis patients (<90 days on hemodialysis) vs the same patients after 6 months of hemodialysis

Aspects of quality of care	Incident patients (<90 days) *n*=258	Prevalent patients (>6 months) *n*=258	p*
	Percent	Percent	Chi square
**Adequacy**			
Weekly treatment time ≥720 min	18	28	<0.001
Blood flow rate ≥300 ml/min	44	71	<0.001
Kt/V >1.2	69	84	<0.001
Treated blood volume ≥1 l/kg	42	67	<0.001
**Nutrition**			
Albumin ≥40 g/l	28	48	<0.001
**Anemia**			
Hb <10 mg/dl	53	26	
Hb 10-12 mg/dl	40	54	<0.001
Hb >12 mg/dl	7	19	
TSAT ≥20%	67	72	0.015
Ferritin ≥200 μg/l	58	72	0.001
**CKD MBD**			
Phosphorus ≤5.5 mg/dl	26	30	NS
Calcium ≤10.2 mg/dl	98	95	NS
PTH 150-600 pg/ml	61	60	NS

### Logistic regression analyses of parameters affecting treatment targets

Table 5 shows logistic regression analyses of parameters influencing hemodialysis adequacy (Table 5A), serum albumin (Table 5B), anemia (Table 5C) and CKD MBD (Table 5D). Women, patients with an increase in treatment time during the six prospective months (∆ treatment time) and patients with a higher BMI were more likely to attain a Kt/V ≥1.2 (Table 5A). Patients with low Charlson comorbidity index were more likely to reach a serum albumin ≥40 g/l (Table 5B) and patients with an increase in TSAT over 6 months (∆TSAT) had significantly higher odds ratio to have a Hb between 10-12 g/dl (Table 5C).

**Table 5:** Logistic (b) regression analysis of effects on the achievement of international treatment targets. A. adequacy (Kt/V >1.2), B. nutrition (albumin >40 g/l), C. anemia (Hb 10-12 g/dl) and D. PTH 150-600 pg/ml

	Odds ratio (CI)	*p*
A. Logistic regression analysis on hemodialysis adequacy (Kt/V ≥1.2)		
Age (years)	1.00 (0.98-1.03)	0.863
Gender (female)	0.42 (0.20-0.910)	0.028
Charlson comorbidity index	0.96 (0.843-1.085)	0.484
BMI (kg/m^2^)	1.08 (1.02-1.14)	0.010
∆Treatment time (min per week)	1.01 (1.00-1.01)	0.003
∆Blood flow (ml/min)	1.01 (0.99-1.01)	0.089
B. Logistic regression analysis on nutrition (albumin ≥40 g/l)		
Age (years)	0.99 (0.97-1.01)	0.261
Gender (female)	0.70 (0.37-1.31)	0.267
Charlson comorbidity index	0.88 (0.776-0.999)	0.049
BMI (kg/m^2^)	0.98 (0.94-1.03)	0.478
∆Treatment time (min per week)	1.00 (0.99-1.003)	0.897
∆Blood flow (ml/min)	0.99 (0.99-1.001)	0.075
∆Kt/V	1.71 (0.81-3.62)	0.161
C. Logistic regression analysis on anemia (Hb 10-12 g/dl)		
Age (years)	1.003 (0.98-1.02)	0.762
Gender (female)	1.24 (0.69-2.60)	0.474
Charlson comorbidity index	1.071 (0.959-1.196)	0.225
∆Blood flow (ml/min)	1.003 (0.998-1.008)	0.223
∆Kt/V	1.08 (0.53-2.20)	0.828
∆TSAT (%)	1.03 (1.01-1.05)	0.017
∆Ferritin (μg/l)	0.99 (0.998-1.000)	0.041
D. Logistic regression analysis on CKD-MBD (iPTH 150-600 pg /ml)		
Age (years)	1.002 (0.99-1.03)	0.869
Gender (female)	0.98 (0.49-1.96)	0.956
Charlson comorbidity index	1.017 (0.91-1.137)	0.767
∆Kt/V	1.10 (0.49-2.50)	0.817
∆Treatment time (min per week)	1.001 (0.998-1.004)	0.403
∆Phosphorus (mg/dl)	0.98 (0.82-1.18)	0.826
∆Calcium (mg/dl)	0.91 (0.73-1.39)	0.408

## Discussion

The annual mortality rate for patients on maintenance hemodialysis is several times higher than that of the general population [1, 16]. Compared to prevalent patients, incident hemodialysis patients experience an even higher risk of mortality within the first few months after initiation of dialysis [2, 3, 16]. Identifying practices and modifiable treatment features that are associated with higher risk of death during this early dialysis period is thus of importance. Some argue that this high-risk period should be one important focus for future clinical investigations [17, 18]. It is important to explore differences in hemodialysis practices and outcomes and to identify optimal treatment approaches to improve early patient survival.

In the present large European multicenter analysis of incident hemodialysis patients, we demonstrate that the use of medical protocols and consistent monthly follow up of laboratory and medical targets to align with recommendations in international guidelines, assures significant improvements in quality of care in many clinical areas, which may correspond to better outcomes.

In the field of chronic kidney disease and dialysis, international clinical practice guidelines have been developed and implemented to improve patient care and outcomes [19–21]. The National Kidney Foundation manages Kidney Disease Outcomes Quality Initiative (KDOQI) [19]. The KDIGO initiative [20] is aimed at improving the care and outcomes of kidney disease patients worldwide, through the development and implementation of global clinical practice guidelines. In parallel, the European Renal Association and the European Dialysis and Transplant Association (ERA EDTA) have initiated the ERBP initiative [21].

The attainment of five guideline targets for hemodialysis patients in European countries (hypertension, anemia, dyslipidemia, metabolic acidosis and CKD-MBD) has recently been audited and revealed to be low overall and far from complete, with substantial differences between countries, which emphasizes the importance of optimizing the care of hemodialysis patients in Europe [22–24]. The EURODOPPS consortium calculated the risk of death and hospital admissions as a function of the simultaneous attainment of clinical guideline targets for hypertension, anemia and CKD-MBD in a large cohort of European dialysis patients [24]. Low attainment of treatment targets was associated with higher risk of mortality, and high fulfilment was independently associated with lower mortality. This association increased gradually as a function of degree of target attainment [24]. Moreover, the risk of early death after starting hemodialysis has been shown to be independently linked with failure to accomplish guideline-based treatment targets for dose of hemodialysis, serum albumin, type of vascular access and level of hemoglobin [10–12].

An analysis of patients on maintenance hemodialysis demonstrated that survival was significantly associated with higher adherence to the clinical targets specified by the KDOQI guidelines [11]. In addition, the dialysis unit practice score, developed as part of the international Dialysis Outcomes and Practice Patterns Study (DOPPS), was strongly linked to outcomes [10–12]. Simultaneous achievement of dialysis dosage, anemia, and serum albumin targets was associated with a marked reduction in mortality and other studies extend these observations to include serum calcium, phosphorus, and PTH targets [25–29]. Patients increasingly meeting more quality goals also report better quality of life [30].

In the present study, both Kt/V and URR were significantly higher in prevalent hemodialysis patients, who had been surveilled in the systematic quality program to attain treatment targets, than in incident patients, which is a consequence of a significant increase in prescribed treatment time, blood flow rate and consequently a higher treated blood volume per session. Logistic regression analysis identified longer treatment time as the most important feature affecting attainment of target dialysis dose, measured as Kt/V.

Previous studies have demonstrated favorable clinical outcomes with longer treatment time and shown associations of longer treatment with better anemia, phosphorus and blood pressure control as well as improved survival among hemodialysis patients [31].

One clinically important finding is the significant improvement in serum albumin from 37 g/l to 41 g/l in patients on hemodialysis for more than three and six months respectively, compared to incident patients. Serum albumin is a strong prognostic factor for adverse outcomes in adults on hemodialysis. Hypoalbuminemia may reflect poor nutrition or presence of inflammation and predicts hospitalization and mortality. Protein energy wasting is closely associated with malnutrition, inflammation and arteriosclerosis and serum albumin is an established surrogate biomarker [32]. This accords with our observation in the present study, showing that patients with low Charlson comorbidity index were more likely to attain this treatment target.

Patients in the current study showed improved anemia control with significantly higher Hb, TSAT and ferritin in combination with significantly lower doses of ESA and lower ERI in prevalent patients compared to incident patients. In addition, the attainment of anemia guidelines was significantly better in prevalent patients. Anemia is associated with morbidity and mortality in chronic kidney disease. The use of ESA is associated with improved functional status, quality of life, and lower requirements for blood transfusion. At the same time, ESAs constitute the largest share of the costs of injectable drugs used in patients on dialysis in many countries. High doses of ESA in patients with comorbidities have however been associated with worse outcomes in randomized controlled trials [33, 34]. The optimal iron management practice to support ESA therapy remains uncertain. In patients on hemodialysis, a high-dose intravenous iron regimen administered proactively was superior to a low-dose regimen administered reactively and not only resulted in lower doses of ESA being administered but also in reduction of cardiovascular end points (non-fatal myocardial infarction, non-fatal stroke and heart failure hospitalization) [35].

In terms of achieving CKD-MBD guidelines targets for PTH in the present study, we observed a significant switch with fewer prevalent patients having a PTH <150 pg/ml and more prevalent patients having PTH >600 pg/ml. Abnormalities in serum calcium, phosphorus, and PTH concentrations are common in patients with chronic kidney disease and have been associated with increased cardiovascular calcification, arterial dysfunction, morbidity and mortality [25–27]. However, no clinical trials have been conducted to clearly identify categories of calcium, phosphorus, and PTH levels associated with the lowest mortality risk. A PTH>600 pg/ml has been associated with adverse clinical outcomes in most published studies and patients with higher PTH had longer duration of dialysis [25–27]. We do not have information on the use of phosphate binders, vitamin D or cinacalcet in the current study.

In the present study, blood pressure was similar in prevalent and incident patients, but IDWG was significantly higher in prevalent patients, potentially reflecting loss of residual urine output between zero and 90 days of hemodialysis. Observational studies have provided conflicting data on the relationships between blood pressure and mortality among hemodialysis patients. Some studies suggest elevated mortality rates at low and not high, blood pressure and other studies have identified a U-shaped correlation between blood pressure and mortality in dialysis [36].

The exact relationship between IDWG and blood pressure control is incompletely characterized. In prevalent hemodialysis subjects, increasing percentage of IDWG is associated with increases in predialysis blood pressure [37].

In this study, we demonstrate a significant increase in the use of AV fistula in prevalent patients (70%) compared to incident patients (32%). Importantly, the use of a central venous catheter for hemodialysis decreased significantly in prevalent patients. The National Kidney Foundation (NKF) recommends an AV fistula as the optimal vascular access due to its higher long-standing patency and lower intervention rate than other vascular access types, which translates into benefits in morbidity and mortality. Hemodialysis guidelines recommend placement of an AV fistula at least six months before the predicted start of hemodialysis, but despite these guidelines, in 2015, 80% of patients in the United States started hemodialysis with a central venous catheter, only 17% with an AV fistula, and only 3% with an AV graft [38].

Satisfying guidelines for multiple parameters is a therapeutic challenge. We observed significant increases in guideline adherence for adequacy, nutrition and vascular access over time. This may reflect in part improved clinical practice patterns and in part survivor bias. Clinical practice patterns associated with increased guideline adherence should be identified and implemented by all providers of dialysis care. Meeting such targets should be viewed as routine and a minimum standard of care for all new patients on dialysis and a complement to personalized, holistic and multidisciplinary team led care approach to individual patients.

The strength of this study is that it included all prevalent patients at all hemodialysis facilities in DaVita centers in Poland and Portugal, thus representing a real-world experience. This study has limitations. We do not have data on hard outcomes, such as hospitalizations and mortality and limited information of prescribed medication. Furthermore, we do not know to what extent incident dialysis patients are intentionally prescribed for example lower treatment times and blood flow rates, resulting in lower Kt/V, due to presence of significant residual kidney function in the first months after start of dialysis. We do not have robust data on the number of incident patients that were transplanted or died during the observation period. In addition, prevalent patients may be a selected group of more healthy survivors and potentially more compliant. Also, in the subgroup of 258 patients followed over time there may be a selection of individuals with lower risk.

## Conclusion

This large European multicenter analysis of incident hemodialysis patients indicates that the use of medical protocols to achieve important clinical targets assures significant improvements in quality of care, which may correspond to better patient outcomes.

## Data Availability

The datasets used and/or analyzed during the current study are available from the corresponding author on reasonable request.

## Abbreviations

BMI: Body mass index
ESA: Erythropoiesis stimulating agent
ESRD: End-stage renal disease
Hb: Hemoglobin
IV: Intravenous
PTH: Parathyroid hormone
SC: Subcutaneous
TSAT: Transferrin saturation

## References

[1] Soucie JM, McClellan WM (1996). Early death in dialysis patients: risk factors and impact on incidence and mortality rates. J Am Soc Nephrol.

[2] Bradbury BD, Fissell RB, Albert JM, Anthony MS, Critchlow CW, Pisoni RL, Port FK, Gillespie BW (2007). Predictors of early mortality among incident US hemodialysis patients in the Dialysis Outcomes and Practice Patterns Study (DOPPS). Clin J Am Soc Nephrol.

[3] Chan KE, Maddux FW, Tolkoff-Rubin N, Karumanchi SA, Thadhani R, Hakim RM (2011). Early outcomes among those initiating chronic dialysis in the United States. Clin J Am Soc Nephrol.

[4] Chaknos CM, Berns JS (2010). Initiating dialysis at the right time: is the evidence IDEAL?. Semin Dial.

[5] Harris A, Cooper BA, Li JJ, Bulfone L, Branley P, Collins JF, Craig JC, Fraenkel MB, Johnson DW, Kesselhut J, Luxton G, Pilmore A, Rosevear M, Tiller DJ, Pollock CA, Harris DC (2011). Cost-effectiveness of initiating dialysis early: a randomized controlled trial. Am J Kidney Dis.

[6] Rosansky S, Glassock RJ, Clark WF (2011). Early start of dialysis: a critical review. Clin J Am Soc Nephrol.

[7] Liberek T, Warzocha A, Galgowska J, Taszner K, Clark WF, Rutkowski B (2011). When to initiate dialysis--is early start always better?. Nephrol Dial Transplant.

[8] Cooper BA, Branley P, Bulfone L, Collins JF, Craig JC, Fraenkel MB, Harris A, Johnson DW, Kesselhut J, Li JJ, Luxton G, Pilmore A, Tiller DJ, Harris DC, Pollock CA, IDEAL Study (2010). A randomized, controlled trial of early versus late initiation of dialysis. N Engl J Med.

[9] Kanda E, Erickson K, Bond TC, Krisher J, McClellan WM (2011). Hemodialysis treatment center early mortality rates for incident hemodialysis patients are associated with the quality of care prior to starting but not following onset of dialysis. Am J Nephrol.

[10] Plantinga LC, Fink NE, Jaar BG, Sadler JH, Levin NW, Coresh J, Klag MJ, Powe NR (2007). Attainment of clinical performance targets and improvement in clinical outcomes and resource use in hemodialysis care: a prospective cohort study. BMC Health Serv Res.

[11] Lacson E, Wang W, Lazarus JM, Hakim RM (2009). Hemodialysis facility-based quality-of-care indicators and facility-specific patient outcomes. Am J Kidney Dis.

[12] Tentori F, Hunt WC, Rohrscheib M, Zhu M, Stidley CA, Servilla K, Miskulin D, Meyer KB, Bedrick EJ, Johnson HK, Zager PG (2007). Which targets in clinical practice guidelines are associated with improved survival in a large dialysis organization?. J Am Soc Nephrol.

[13] Stack AG (2003). Impact of timing of nephrology referral and pre-ESRD care on mortality risk among new ESRD patients in the United States. Am J Kidney Dis.

[14] Lorenzo V, Martin M, Rufino M, Hernández D, Torres A, Ayus JC (2004). Predialysis nephrologic care and a functioning arteriovenous fistula at entry are associated with better survival in incident hemodialysis patients: an observational cohort study. Am J Kidney Dis.

[15] Hasegawa T, Bragg-Gresham JL, Yamazaki S, Fukuhara S, Akizawa T, Kleophas W, Greenwood R, Pisoni RL (2009). Greater first-year survival on hemodialysis in facilities in which patients are provided earlier and more frequent pre-nephrology visits. Clin J Am Soc Nephrol.

[16] Noordzij M, Jager KJ (2014). Increased mortality early after dialysis initiation: a universal phenomenon. Kidney Int.

[17] Khan SS, Xue JL, Kazmi WH, Gilbertson DT, Obrador GT, Pereira BJ, Collins AJ (2005). Does predialysis nephrology care influence patient survival after initiation of dialysis?. Kidney Int.

[18] Nee R, Fisher E, Yuan CM, Agodoa LY, Abbott KC (2017). Pre-end-stage renal disease care and early survival among incident dialysis patients in the US Military Health System. Am J Nephrol.

[19] National Kidney Foundation (2015). KDOQI clinical practice guideline for hemodialysis adequacy: 2015 update. Am J Kidney Dis.

[20] Chan CT, Blankestijn PJ, Dember LM, Gallieni M, Harris DCH, Lok CE, Mehrotra R, Stevens PE, Yee-Moon Wang A, Cheung M, Wheeler DC, Winkelmayer WC, Pollock CA (2019). Dialysis initiation, modality choice, access, and prescription: conclusions from a Kidney Disease: Improving Global Outcomes (KDIGO) Controversies Conference. Kidney Int.

[21] Tattersall J, Martin-Malo A, Pedrini L, Basci A, Canaud B, Fouque D, Haage P, Konner K, Pizzarelli P, Tordoir J, Vennegoor M, Wanner C, Wee P, Vanholder R (2007). EBPG guideline on dialysis strategies. Nephrol Dial Transplant.

[22] Liabeuf S, Van Stralen KJ, Caskey F, Tentori F, Pisoni RL, Sajjad A, Jager KJ, Massy ZA (2017). Attainment of guideline targets in EURODOPPS haemodialysis patients: are differences related to a country’s healthcare expenditure and nephrologist workforce?. Nephrol Dial Transplant.

[23] Hecking E, Bragg-Gresham JL, Rayner HC, Pisoni RL, Andreucci VE, Combe C, Greenwood R, McCullough K, Feldman HI, Young EW, Held PJ, Port FK (2004). Haemodialysis prescription, adherence and nutritional indicators in five European countries: results from the Dialysis Outcomes and Practice Patterns Study (DOPPS). Nephrol Dial Transplant.

[24] Liabeuf S, Sajjad A, Kramer A, Bieber B, McCullough K, Pisoni R, Caskey F, Combe C, Robinson BM, Jager KJ, Massy ZA (2019). Guideline attainment and morbidity/mortality rates in a large cohort of European haemodialysis patients (EURODOPPS). Nephrol Dial Transplant.

[25] Floege J, Kim J, Ireland E (2011). Serum iPTH, calcium and phosphate, and the risk of mortality in a European haemodialysis population. Nephrol Dial Transplant.

[26] Cozzolino M, Messa P, Brancaccio D, Cannella G, Bolasco P, Di Luca M, Costanzo AM, di Luzio PU, Festa V, Gualberti G, Mazzaferro S (2014). Achievement of NKF/K-DOQI recommended target values for bone and mineral metabolism in incident hemodialysis patients: results of the FARO-2 cohort. Blood Purif.

[27] Tangri N, Wagner M, Griffith JL (2011). Effect of bone mineral guideline target achievement on mortality in incident dialysis patients: an analysis of the United Kingdom Renal Registry. Am J Kidney Dis.

[28] Danese MD, Belozeroff V, Smirnakis K, Rothman KJ (2008). Consistent control of mineral and bone disorder in incident hemodialysis patients. Clin J Am Soc Nephrol.

[29] Locatelli F, Pisoni RL, Combe C, Bommer J, Andreucci VE, Piera L, Greenwood R, Feldman HI, Port FK, Held PJ (2004). Anaemia in haemodialysis patients of five European countries: association with morbidity and mortality in the Dialysis Outcomes and Practice Patterns Study (DOPPS). Nephrol Dial Transplant.

[30] Lacson E, Jianglin Xu J, Lin SF, Dean SG, Lazarus JM, Hakim R (2009). Association between achievement of hemodialysis quality-of-care indicators and quality-of-life scores. Am J Kidney Dis.

[31] Tentori F, Zhang J, Li Y, Karaboyas A, Kerr P, Saran R, Bommer J, Port F, Akiba T, Pisoni R, Robinson B (2012). Longer dialysis session length is associated with better intermediate outcomes and survival among patients on in-center three times per week hemodialysis: results from the Dialysis Outcomes and Practice Patterns Study (DOPPS). Nephrol Dial Transplant.

[32] Hara H, Nakamura Y, Hatano M, Iwashita T, Shimizu T, Ogawa T, Kanozawa K, Hasegawa H (2018). Protein energy wasting and sarcopenia in dialysis patients. Contrib Nephrol.

[33] Besarab A, Bolton WK, Browne JK, Egrie JC, Nissenson AR, Okamoto DM (1998). The effects of normal as compared with low hematocrit values in patients with cardiac disease who are receiving hemodialysis and epoetin. N Engl J Med.

[34] Singh AK, Szczech L, Tang KL, Barnhart H, Sapp S, Wolfson M, CHOIR Investigators (2006). Correction of anemia with epoetin alfa in chronic kidney disease. N Engl J Med.

[35] Macdougall IC, White C, Anker S, Bhandari, Farrington K, Kalra PA, McMurray JJV, Murray H, Tomson CRV, Wheeler DC, Winearls CG, Ford I, I for the PIVOTAL Investigators and Committees (2019). N Engl J Med.

[36] Miskulin DC, Weiner DE (2017). Blood pressure management in hemodialysis patients: What we know and what questions remain. Semin Dial.

[37] Ipema KJ, Kuipers J, Westerhuis R, Gaillard CA, van der Schans CP, Krijnen WP, Franssen CF (2016). Causes and consequences of interdialytic weight gain. Kidney Blood Press Res.

[38] Pisoni RL, Zepel L, Port FK, Robinson BM (2015). Trends in US Vascular access use, patient preferences, and related practices: an update from the US DOPPS practice monitor with international comparisons. Am J Kidney Dis.

